# Practice patterns for chronic hypoparathyroidism: data from patients and physicians in France

**DOI:** 10.1530/EC-21-0350

**Published:** 2021-12-23

**Authors:** Jean-Philippe Bertocchio, Natalie Grosset, Lionel Groussin, Peter Kamenický, Fabrice Larceneux, Anne Lienhardt-Roussie, Agnès Linglart, Gérard Maruani, Eric Mirallie, François Pattou, Riyad N H Seervai, Coralie Sido, Caroline Silve, Aurélie Vilfaillot, Antoine Tabarin, Marie-Christine Vantyghem, Pascal Houillier, the investigators of the Épi-Hypo study

**Affiliations:** 1 Assistance Publique-Hôpitaux de Paris, Hôpital Européen Georges Pompidou, Service de Physiologie, Paris, France; 2 Centre de Référence des Maladies Rares du Calcium et du Phosphore Filière de Santé Maladies Rares OSCAR, Paris, France; 3 Centre de Recherche des Cordeliers, INSERM, Sorbonne Université, Université de Paris, INSERM, UMRS1138, Paris, France; 4 Hypoparathyroïdisme France, Annecy, France; 5 Department of Endocrinology, Assistance Publique-Hôpitaux de Paris, Hôpital Cochin, Université de Paris, Paris, France; 6 Université Paris-Saclay, Inserm U1185, Physiologie et Physiopathologie Endocriniennes, Assistance Publique-Hôpitaux de Paris, Hôpital Bicêtre, Service d’Endocrinologie et des Maladies de la Reproduction, Centre de Référence des Maladies Rares du Métabolisme du Calcium et du Phosphate, Le Kremlin-Bicêtre, France; 7 Université Paris-Dauphine, PSL Research University, CNRS, UMR 7088, DRM [Ermes], Paris, France; 8 CHU Dupuytren, Hôpital Mère Enfant, Endocrinologie Pédiatrique, Limoges, France; 9 Université Paris-Saclay, Inserm U1185, Physiologie et Physiopathologie Endocriniennes, Assistance Publique-Hôpitaux de Paris, Service d’Endocrinologie et Diabète de l’Enfant, Centre de Référence des Maladies Rares du Calcium et du Phosphore et Filière de Santé Maladies Rares OSCAR, Hôpital Bicêtre Paris Saclay, Le Kremlin-Bicêtre, France; 10 Assistance Publique-Hôpitaux de Paris, Institut Necker-Enfants Malades, INSERM U1151 – CNRS UMR 8253, Paris, France; 11 Chirurgie Cancérologique, Digestive et Endocrine, Institut des Maladies de l’Appareil Digestif, Hôtel Dieu, CHU Nantes, France; 12 Association Francophone de Chirurgie Endocrinienne (AFCE), France; 13 Université de Lille, CHU Lille, Institut Pasteur Lille, Inserm U1190, Lille, France; 14 Molecular & Cellular Biology Graduate Program, Medical Scientist Training Program, Baylor College of Medicine, Houston, Texas, USA; 15 Assistance Publique-Hôpitaux de Paris, Hôpital Cochin, Biochimie et Génétique Moléculaires, Paris, France; 16 INSERM, U1169, Université Paris Sud, Hôpital Bicêtre, Le Kremlin Bicêtre, France; 17 Assistance Publique-Hôpitaux de Paris, Hôpital Européen Georges Pompidou, Unité de Recherche Clinique, Paris, France; 18 INSERM, U1418, CIC-EC, Hôpital Européen Georges Pompidou, Paris, France; 19 Service Endocrinologie Diabète et Nutrition, CHU de Bordeaux, Université de Bordeaux, Pessac, France; 20 CHU Lille, Department of Endocrinology, Diabetology and Metabolism, Inserm U1190, EGID, Lille, France; 21 CNRS, ERL8228, Paris, France

**Keywords:** hypoparathyroidism, practice patterns, physicians, epidemiological studies, hypocalcemia

## Abstract

**Context:**

Recent guidelines have provided recommendations for the care of patients with chronic hypoparathyroidism. Very little is known about actual physicians’ practices or their adherence to such guidelines.

**Objective:**

To describe the physicians’ practice patterns and their compliance with international guidelines.

**Design:**

The cohort studies included were Épi-Hypo (118 physicians and 107 patients, from September 2016 to December 2019) and ePatients (110 patients, November 2019).

**Methods:**

Internet-based cohorts involving all settings at a nationwide level (France). Participants were (i) physicians treating patients with chronic hypoparathyroidism and patients with chronic hypoparathyroidism either participating in the (ii) Épi-Hypo study (Épi-Hypo 2019 patients), or (iii) Hypoparathyroidism France, the national representative association (ePatients).

**Results:**

The physicians’ specialties were mainly endocrinology (61%), nephrology (28%), family medicine (2.5%), pediatrics (2.5%), rheumatology (2%), or miscellaneous (4%) and 45% were practicing in public universities. The median number of pharmaceutical drug classes prescribed was three per patient. The combination of active vitamin D and calcium salt was given to 59 and 58% of ePatients and Épi-Hypo 2019 patients, respectively. Eighty-five percent of ePatients and 87% of physicians reported monitoring plasma calcium concentrations at a steady state at least twice a year. In 32 and 26% of cases, respectively, ePatients and physicians reported being fully in accordance with international guidelines that recommend targeting symptoms, plasma calcium and phosphate values, and urine calcium excretion.

**Conclusions:**

The care of patients with chronic hypoparathyroidism involves physicians with very different practices, so guidelines should include and target other specialists as well as endocrinologists. Full adherence to the guidelines is low in France.

## Introduction

Hypoparathyroidism is a rare condition caused by undetectable or inappropriately low secretion of parathyroid hormone (PTH) that is insufficient for maintaining the plasma calcium concentration (PCa) within the normal range (1). The most frequent cause is surgical removal (or ischemia) of the parathyroid glands during a parathyroidectomy (e.g. for hyperparathyroidism) or a thyroidectomy (e.g. for thyroid cancer or goiter). Other causes include inherited genetic or chromosomal disorders, as well as infiltrative or autoimmune diseases.

Symptoms (such as paresthesia, cramps or a seizure) are nonspecific, and hypoparathyroidism can be asymptomatic. The diagnosis can be straightforward when symptoms occur within hours to days after neck surgery; however, diagnosis is sometimes delayed for years, especially in children. Diagnosing hypoparathyroidism is crucial due to its renal (nephrolithiasis, nephrocalcinosis, and chronic kidney failure), ocular (cataract), and neurological (brain development alterations, basal ganglia calcifications) effects and its resultant negative impact on the quality of life.

In contrast to other hormone-deficiency syndromes commonly treated by hormone replacement, the standard of care of hypoparathyroidism is based on oral calcium supplementation (Ca salts) and active vitamin D analog (active vitamin D) administration (2, 3). In 2015 and 2016, the European Society of Endocrinology (4) and the First International Conference for the Management of Hypoparathyroidism (5) published guidelines for the diagnosis, treatment, and follow-up of patients with chronic hypoparathyroidism. More recently, the American Thyroid Association (6) and a consensus group (7) also published a series of statements to standardize the care of hypoparathyroidism in light of evidence-based recommendations and to raise issues and interest in future investigations. None of these initiatives fully addresses the management of children with hypoparathyroidism.

Due to the huge phenotypic variability of hypoparathyroidism, physicians involved in the care and follow-up of patients range from pediatricians to endocrinologists and work in very different settings and from family medicine offices to tertiary care hospitals. To increase awareness of the guidelines, it is important to target the right audience (8), but very little is known about who these physicians are and what their daily practice is regarding the treatment and follow-up of patients with chronic hypoparathyroidism. The present study aimed to address this point. We analyzed practice pattern data with the following objectives: (i) to describe the profile of physicians along with their practice pattern in terms of treatment and follow-up, (ii) to identify their objectives for care and follow-up, and (iii) to study the strategies used to meet them. To do so, we collected data from physicians and assessed their concordance with data collected from patients. Combining three different sources of data (one from physicians and two from patients), we were able to evaluate the agreement of their clinical practice with international guidelines.

## Methods

### Data sources and collection

Épi-Hypo (NCT02838927) is a cohort study that aimed to describe the natural history of chronic hypoparathyroidism in France. Data were collected online via a secure website. Physicians had to practice in France and provide care to one or more patient(s) with chronic hypoparathyroidism. They were solicited through national professional societies. At the time of registration, physicians were requested to fill out an online questionnaire (Supplementary material, see section on supplementary materials given at the end of this article) regarding how they monitor and provide care for patients with chronic hypoparathyroidism; the data were collected from September 2016 to November 2019 and are presented as the ‘Physicians data’. Of note, two items (assessment of brain calcifications and bone mineral density) were added later to the study (May 2019); therefore, as mentioned, these data from a subset of physicians are missing. We developed another online questionnaire (Supplementary material) aimed to describe the practice patterns as reported by patients (patient-reported outcomes). Volunteers registered online (therefore called ‘ePatients’) following an invitation sent by the *Hypoparathyroidisme France* association to its members. We collected ‘ePatients data’ during November 2019 and excluded those who also participated in Épi-Hypo. Finally, we also used data collected by investigators from the medical records of patients participating in Épi-Hypo who had at least one follow-up visit in 2019 (referred to as ‘Épi-Hypo 2019 patients’) to obtain data from the same period of time as that of the ePatients.

Chronic hypoparathyroidism was defined as per the latest guidelines (4, 5, 7): the association of hypocalcemia and inappropriately low concentrations of circulating PTH for at least 6 months. The inclusion criteria for patients in Épi-Hypo were as follows: chronic hypoparathyroidism, excluding pseudohypoparathyroidism and transient hypoparathyroidism, and follow-up by a physician in France. All patients gave their informed consent to participate in this study. For patients <18 years old, written consent of one parent was also required. The Épi-Hypo study was conducted in compliance with the Declaration of Helsinki and was approved by an independent ethics committee (CERHUPO 2016-09-05), as well as by the French regulatory board (CNIL no. 916031).

### Statistical analyses

As no assumption could be made on the Gaussian distribution of most data (Shapiro–Wilk test), all data are reported as their median (IQR) or as numbers and proportions (%) for quantitative and qualitative data, respectively. Values were compared by Mann–Whitney or Kruskal–Wallis tests (with Dunn’s multiple comparison test) when more than two groups were compared, or chi-square tests where appropriate using RStudio: Integrated Development Environment for R (RStudio, Boston, MA, USA; http://www.rstudio.com/). Venn diagrams were designed using a publicly available website (http://bioinformatics.psb.ugent.be). We considered a *P* -value <0.05 to be significant in all cases.

## Results

### Demographics

**Figure 1:**
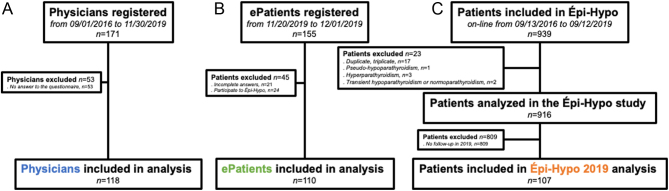
Flowcharts of the analyzed cohorts. (A) From September 2016 to November 2019, 171 physicians were enrolled in the Épi-Hypo study. Among them, 118 (69%) answered the questionnaire about their habits in terms of follow-up of patients with chronic hypoparathyroidism; their data are presented as ‘Physicians’ data. (B) In November 2019, we conducted an online questionnaire dedicated to patients living with chronic hypoparathyroidism. We collected 155 answers. Among them, 110 (71%) were analyzed; we present that data as the ‘ePatients’ data. (C) From September 2016 to September 2019, 939 patients were enrolled in the Épi-Hypo study. Among them, 916 (98%) met the inclusion criteria. Finally, 107 (12%) had a follow-up visit in 2019; we present that data as the ‘Épi-Hypo2019’ data.

Out of the 171 physicians who registered to Épi-Hypo, we analyzed the data from the 118 (69%) physicians who completed their questionnaire (Fig. 1 and Table 1). Most of the physicians were female (63%), endocrinologists (61%), or nephrologists (28%), and almost half (45%) were practicing in public universities. Out of them, 93 (79%) and 90 (76%) stated that their initial or continuous medical education, respectively, were not sufficient to take care of such patients.

**Table 1:** Characteristics of the physicians. The data were extracted from the Épi-Hypo study to determine the practices of clinicians (physicians). Data are presented as medians (IQRs) and numbers (percentages), as appropriate.

	Physicians, *n* = 118
Gender (female), *n* (%)	74 (63)
Age, years	41.0 (35.0–54.0)
Specialty, *n* (%)	
Endocrinology	72 (61)
Nephrology	33 (28)
Family medicine	3 (2.5)
Pediatrics	3 (2.5)
Rheumatology	2 (2)
Other	5 (4)
Structure, *n* (%)	
For-profit private	22 (19)
Public university	53 (45)
Public non-university	23 (19)
Non-profit private	20 (17)

We then analyzed the two cohorts of patients: first, the data of 107 Épi-Hypo 2019 patients; secondly, the data of 110 patients who participated in the ePatients survey. ePatients were more frequently female (91% vs 71%) and were slightly younger (45 vs 51 years old) than Épi-Hypo 2019 patients (Table 2), which is a pattern known for e-surveys (9). Their duration of hypoparathyroidism was shorter than that of Épi-Hypo 2019 patients (5.0 vs 10.0 years), whereas the delay to diagnosis was similar. We collected data from two children, in ePatients only. The various causes of hypoparathyroidism did not differ between the two cohorts of patients, with surgery being the most prominent cause. Two patients (belonging to the Épi-Hypo 2019 patients) had end-stage kidney disease (one treated by hemodialysis and one by kidney transplantation). The medical specialty of physicians following up with patients in both cohorts differed, as more endocrinologists were involved in the care and follow-up of ePatients (65% *vs.*57%), whereas more nephrologists were involved in the care and follow-up of Épi-Hypo 2019 patients (42% *vs.*10%). ePatients were more frequently followed in a for-profit office than Épi-Hypo 2019 patients (51% vs 8%); physicians working in a public university hospital followed 38% of ePatients and 75% of Épi-Hypo 2019 patients.

**Table 2:** Characteristics of the ePatients and Épi-Hypo 2019 cohorts. The data were extracted from the Épi-Hypo study to determine the characteristics of patients who had at least one follow-up visit in 2019 (Épi-Hypo 2019). We compared these data to those obtained from an online survey conducted in patients with chronic hypoparathyroidism (ePatients). Data are presented as medians (IQRs) and numbers (percentages), as appropriate.

	Overall, *n* = 217	ePatients, *n* = 110	Épi-Hypo 2019, *n = *107	*P*	SMD
Gender (female), *n* (%)	176 (81.1)	100 (90.9)	76 (71.0)	<0.001	0.524
Age, years	48.0 (38.0, 57.0)	45.0 (36.5, 54.0)	51.0 (38.0–63.0)	0.023	0.348
<18, *n* (%)	2 (0.9)	2 (1.8)	0 (0.0)	0.49	0.192
Physicians					
Specialty, *n* (%)				<0.001	1.059
Endocrinology	132 (60.8)	71 (64.5)	61 (57.0)		
Nephrology	56 (25.8)	11 (10.0)	45 (42.1)		
Family medicine	24 (11.1)	24 (21.8)	0 (0.0)		
Pediatrics	0 (0.0)	0 (0.0)	0 (0.0)		
Rheumatology	1 (0.5)	1 (0.9)	0 (0.0)		
Other	4 (1.8)	3 (2.8)	1 (0.9)		
Structure^a^, *n* (%)				<0.001	1.145
For-profit private	63 (29.3)	55 (50.0)	8 (7.5)		
Public university	121 (56.3)	41 (37.3)	80 (74.8)		
Public non-university	19 (8.8)	10 (9.1)	9 (8.4)		
Non-for-profit private	12 (5.6)	2 (1.8)	10 (9.3)		
Hypoparathyroidism					
Duration, years	7.00 (3.00, 15.00)	5.00 (2.00, 13.50)	10.00 (6.00, 15.00)	<0.001	0.412
Delay to diagnosis, years	0.00 (0.00, 1.00)	0.00 (0.00, 1.00)	0.00 (0.00, 1.50)	0.303	0.096
Cause, *n* (%)				0.131	0.275
Surgery	170 (78.3)	91 (82.7)	79 (73.8)		
Inherited	20 (9.2)	6 (5.5)	14 (13.1)		
Other	27 (12.5)	13 (11.8)	14 (13.1)		

^a^Data are missing for two ePatients.

SMD, standardized mean difference.

### Therapeutics

**Figure 2:**
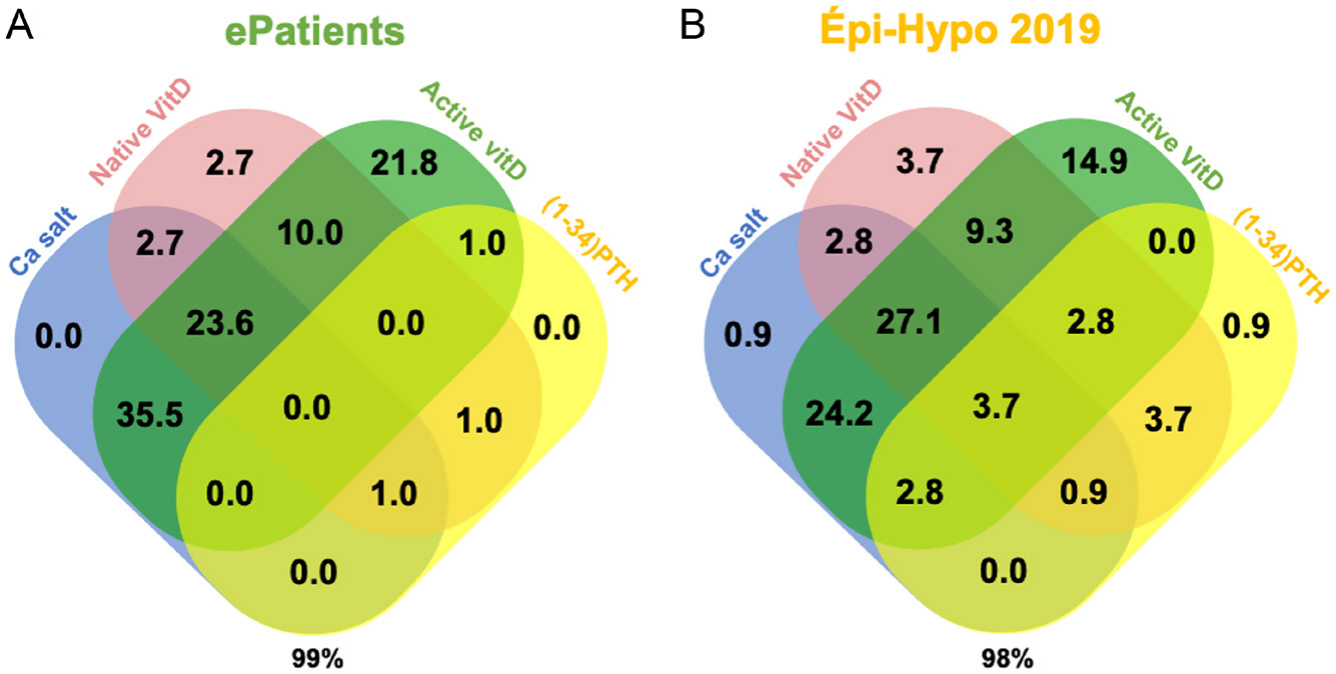
Combinations of treatment during chronic hypoparathyroidism. (A) Among 110 patients in the ePatients cohort, 109 (99%) were treated with an oral calcium supplement (Ca salt), native vitamin D (native vitamin D), active analogs of vitamin D (active vitamin D), and/or teriparatide (PTH(1–34)). (B) Among 107 patients of the Épi-Hypo 2019 cohort, 105 (98%) were treated with one or more of those therapeutic classes. Data are presented as percentages (%) of the total answers.

The most frequently prescribed drug classes were active vitamin D, with a predominance of alfacalcidol over calcitriol and Ca salts (Table 3). The percentages of ePatients and Épi-Hypo 2019 patients treated with Ca salts, active vitamin D, and thiazide diuretics were similar. Native vitamin D (native vitamin D) (40.9% *vs.*55.1%) and teriparatide (2.7% *vs.*15.0%) were less frequently reported by ePatients than Épi-Hypo 2019 patients, whereas magnesium supplements were more frequently reported in ePatients than in Épi-Hypo 2019 patients (40% vs 23%). The median number of pharmacological classes per patient was 3 for both ePatients and Épi-Hypo 2019 patients. The combination of pharmacological classes was similar in both cohorts of patients (Fig. 2); all of the possible combinations of Ca salt, native vitamin D, active vitamin D, and teriparatide (PTH(1–34)) accounted for 98–99% of the possibilities in both populations. The combination of active vitamin D and Ca salt (with or without native vitamin D) was given to 59.1 and 57.8% of ePatients and Épi-Hypo 2019 patients, respectively. Five (4.5%) and 11 (10.3%) patients did not receive any Ca salt or active vitamin D in the ePatients and Épi-Hypo 2019 cohorts, respectively; these proportions were similar (*P*  = 0.12). Overall, the treatment of hypoparathyroidism in the ePatients and Épi-Hypo 2019 cohorts was qualitatively similar, except for magnesium supplements, native vitamin D, and teriparatide (PTH(1–34)).

**Table 3:** Pharmacological classes prescribed for the care of patients with chronic hypoparathyroidism in the ePatients and Épi-Hypo 2019 cohorts. The data were extracted from the Épi-Hypo study to determine the prescriptions of patients who had at least one follow-up visit in 2019 (Épi-Hypo 2019). We compared the data to those obtained from an online survey performed in patients with chronic hypoparathyroidism (ePatients). Native vitamin D refers to cholecalciferol or ergocalciferol, regardless of the formulation. Data are presented as medians (IQRs) and numbers (percentages), as appropriate.

	Overall, *n* = 217	ePatients, *n* = 110	Épi-Hypo 2019, *n* = 107	*P*	SMD
Number of pharmacological classes	3.0 (2.0, 4.0)	3.0 (3.0, 4.0)	3.0 (2.0, 3.0)	<0.001	0.744
Calcium salt, *n* (%)	136 (62.7)	69 (62.7)	67 (62.6)	1	0.002
Native vitamin D, *n* (%)	104 (47.9)	45 (40.9)	59 (55.1)	0.042	0.288
Alfacalcidol, *n* (%)	163 (75.1)	84 (76.4)	79 (73.8)	0.754	0.059
Calcitriol, *n* (%)	29 (13.4)	17 (15.5)	12 (11.2)	0.427	0.125
Magnesium salt, *n* (%)	69 (31.8)	44 (40.0)	25 (23.4)	0.009	0.363
Thiazide diuretics, *n* (%)	26 (12.0)	11 (10.0)	15 (14.0)	0.408	0.124
Non-calcium-based phosphate binder, *n* (%)	6 (2.8)	1 (0.9)	5 (4.7)	0.116	0.230
Teriparatide (PTH(1–34)), *n* (%)	19 (8.8)	3 (2.7)	16 (15.0)	0.001	0.441

SMD, standardized mean difference.

### Monitoring

**Figure 3:**
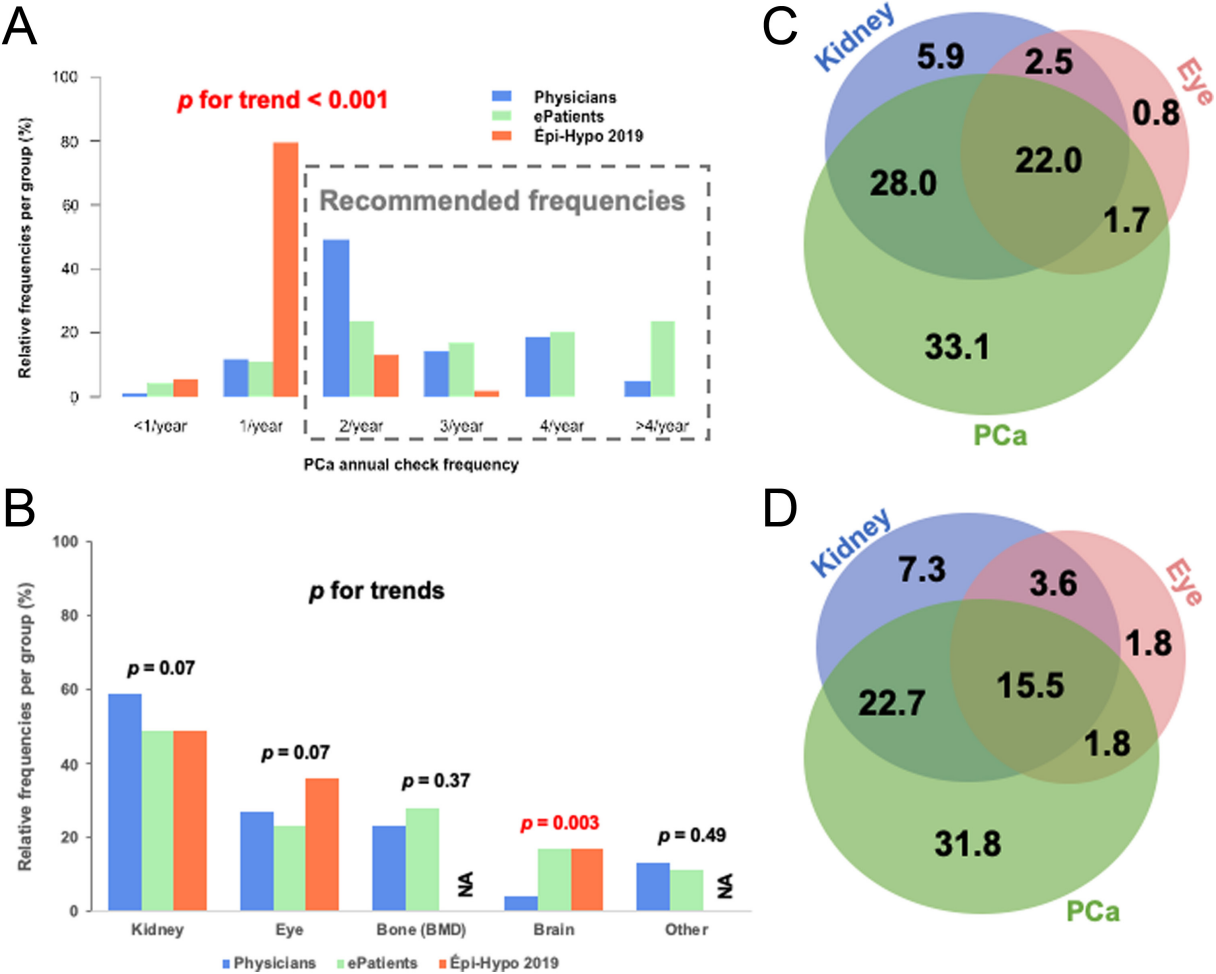
Trends in follow-up habits during chronic hypoparathyroidism. (A) Data from the ‘Physicians’ cohort (blue, *n* = 118) show that they are more akin to checking plasma calcium (PCa) twice a year, while the ePatients (green) report a broader distribution (more frequent checks), and data from the Épi-Hypo 2019 (orange, *n* = 107) cohort report less frequent measurements of PCa (*P* < 0.001). In the ePatients (green) who reported screening for PCa (n  = 93), the distribution was broader. (B) The most frequently checked organs during chronic hypoparathyroidism are the kidneys, eyes, bone (by bone mineral density, BMD), and brain. Some ePatients and physicians also reported other organs (such as the heart) that could be checked. Of note, no data (NA) are available regarding bone or other follow-up from the Épi-Hypo 2019 cohort, and because the questions were added later, only 35 and 26 physicians answered whether they screened for BMD and brain, respectively. (C) Venn diagram showing the combinations of usual follow-up during chronic hypoparathyroidism by physicians: among the 118 physicians of the cohort, 111 (94%) follow kidney imagery, ask for eye checks, and/or checked PCa (at least twice a year). (D) Venn diagram showing the combinations of usual follow-up during chronic hypoparathyroidism reported by ePatients; among the 110 patients of the cohort, 93 (85%) were followed with kidney imagery, asked for eye checks, and/or checked for PCa (at least twice a year). Data are presented as percentages (%) of the total answers.

Our purpose was to outline the monitoring of patients with chronic hypoparathyroidism while on ‘steady state’, excluding periods of rapid fluctuations in PCa (Fig. 3). Forty-nine percent of the 118 physicians requested a PCa measurement twice a year (Fig. 3A). Forty-four percent of the 93 ePatients who had their PCa measured reported a frequency of more than thrice a year. In 2019, the investigators of the Épi-Hypo study collected one PCa value in 79% of patients and two values in 13% of patients, which differs (*P*  < 0.001 for trend) from what was reported by physicians (12 and 49%, respectively) and ePatients (11 and 24%, respectively). International guidelines recommend monitoring PCa at least twice a year at steady state. Therefore, 87% of physicians and 85% of ePatients report proceeding accordingly.

Screening for nephrolithiasis and nephrocalcinosis was carried out in 49% of ePatients, in 49% of Épi-Hypo 2019 patients, and was requested by 59% of physicians (Fig. 3B). Of them, 90% used ultrasonography and 72% of ePatients whom kidney morphology was analyzed had ultrasonography performed. Urine calcium excretion was measured at least once a year for 85 and 53% of ePatients and Épi-Hypo 2019 patients, respectively. Screening for ocular complications (cataracts) was carried out in 23% of ePatients, in 36% of Épi-Hypo 2019 patients, and was requested by 27% of physicians. Monitoring of bone mineral density was reported by 28% of ePatients and 23% of physicians. Even though few physicians reported their practice regarding basal ganglia calcification monitoring, five (4%) of them reported following-up brain complications, which is a lower proportion than that reported by ePatients (17%) and the one observed in Épi-Hypo 2019 patients (17%).

Figure 3C and  D summarizes the various combinations of items monitored, as reported by physicians (Fig. 3C) and ePatients (Fig. 3D). In 32 and 33% of ePatients and physicians, respectively, PCa was the sole item that was monitored. Seven percent of physicians and 6% of ePatients indicated that kidney morphology was the only item monitored; 38% of ePatients and 50.0% of physicians declared that both PCa and kidney morphology were monitored.

### Objective(s) of treatment

**Figure 4:**
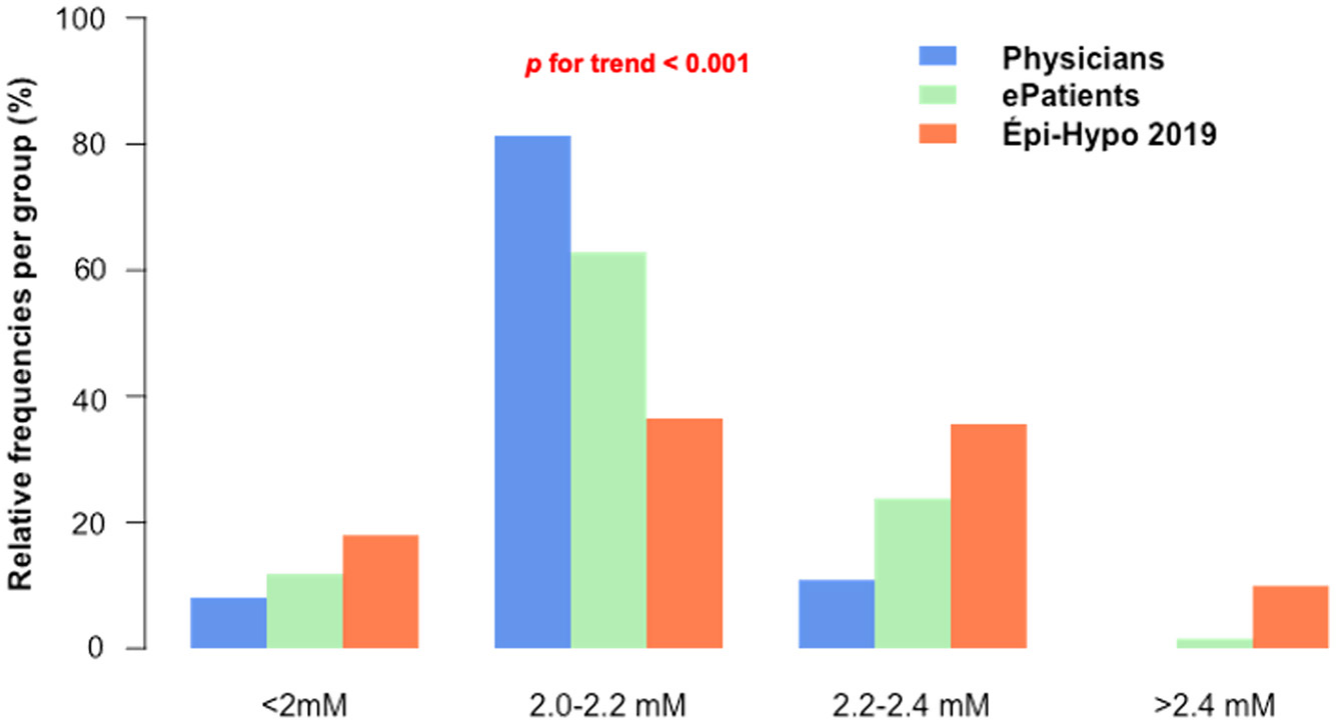
Plasma calcium targets during chronic hypoparathyroidism and values reached. While physicians (blue) and ePatients (green) report a targeted plasma calcium concentration mainly in the lowest range of the normal values (2.0–2.2 mM), the values observed (i.e. measured) in the Épi-Hypo study (orange) show a broader distribution that is significantly higher.

**Figure 5:**
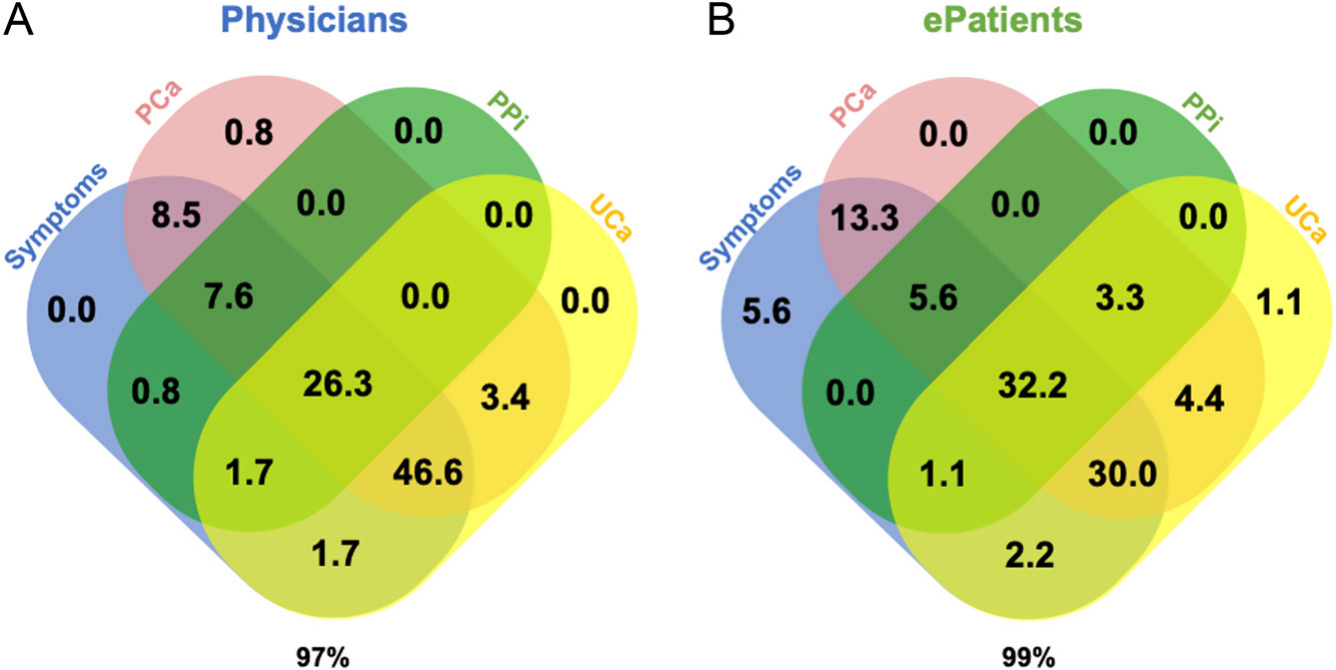
Indicators used as targets in the treatment during chronic hypoparathyroidism. (A) Venn diagram showing the combinations of indicators used by physicians: among the 118 included physicians, 115 (97%) targeted symptoms, plasma calcium (PCa), plasma phosphate concentration (PPi), and/or calciuria (UCa). (B) Venn diagram showing the combinations of indicators used by ePatients: among the 90 included ePatients who knew the indicator(s) of their treatment, 89 (99%) reported symptoms, PCa, PPi, and/or UCa as indicators. Of note, the combination PCa-UCa accounted for 76 and 70% of cases in the physicians and ePatients data, respectively. Data are presented as percentages (%) of the total answers.

The purpose of treating patients is to provide them with better health. To achieve this purpose, physicians have to follow specific indicators and to target specific objectives (values for numerical indicators or states for others). Here, we studied five specific indicators: clinical symptoms, PCa value, plasma phosphate concentration (PPi) value, calcium–phosphate product value, and urine calcium excretion (UCa) value. The absence of symptoms was an objective for 113 physicians (96%). Moreover, 112 (95%) reported that the PCa value was an indicator, and among them, 91 (81%) had a target interval in the low normal range (2.0–2.2 mM, 8.0–8.8 mg/dL, Fig. 4). The PPi value was an indicator for 43 physicians (36%); the objective was a PPi value within the normal range. The plasma calcium–phosphate product value was an indicator for 10 physicians (8%); the objective was a value below 4.4 mmol^2^/L^2^ (Fig. 5A). Finally, UCa was an indicator for 97 physicians (82%), of whom 87 (90%) used 24-h urine collection to measure it; their target was a 24-h UCa value below 10 mmol/day for 65 (75%) of them. Overall, 97% of physicians reported that symptoms, PCa, PPi, and/or UCa were indicators.

Among the 110 ePatients, 90 (82%) reported that they were aware of the indicators used by their physician. Overall, 99% of them reported that symptoms, PCa, PPi, and/or UCa were used as indicators by their physician. Eighty ePatients (89%) reported that the PCa value was an indicator and 42 of them (53%) reported that the low-normal range of PCa was the target (Fig. 4). Symptoms, PPi value, calcium–phosphate product, and UCa were reported as indicators by 81 (90%), 38 (42%), 12 (13%), and 67 (74%) of these 90 ePatients (Fig. 5B). In addition to PCa, no data were specifically recorded regarding the target values.

From the Épi-Hypo 2019 cohort, we found that 101 (94%) patients had a PCa measured during this period; out of them, 33 (33%) had a value between 2.0 and 2.2 mM. This proportion highly differed (*P*  < 0.001) from the objectives of ePatients and physicians, among whom 81 and 53% reported targeting this specific range (Fig. 4). We thus conclude that the measured PCa values differ considerably from the generally targeted values.

Figure 5 summarizes the various combinations of indicators, as reported by physicians (Fig. 5A) and ePatients (Fig. 5B). The most frequently combined indicators were PCa and UCa, with similar frequencies in the ePatients (66%) and physicians (76%) cohorts. Considering international guidelines recommending targeting symptoms, PCa, PPi, and UCa, only 31 (26%) physicians and 29 (32%) ePatients were fully in agreement with the guidelines.

## Discussion

Hypoparathyroidism is a rare disease that can afflict newborns, infants, children, and adults: this large age range increases the diversity of caregivers and the range of information that guidelines need to deliver. The first aim of our work was to investigate who the healthcare providers are. We report that endocrinologists, although the most frequently involved, are part of a large network of physicians. This contrasts with a previous US study in which 90% of patients were followed by endocrinologists (10). The lower rate that we observed in France is based on data from two independent sources: one physician-based and another patient-based that we used as an external validation cohort. It could be that French and American healthcare systems have different patterns or that the former publication only reported data from a selected population followed in a tertiary care hospital.

This point is especially important regarding the cause of hypoparathyroidism. Mitchell *et al.*reported that 60% of cases were postsurgical – which is similar to findings by other tertiary-care hospitals (11) – while we found a much larger proportion (74–84%), closer to data from population-based cohorts (12). The difference could be explained by various definitions of chronic hypoparathyroidism (13); some can include pseudohypoparathyroidism (11) or only ‘PTH level below the lower limit of the laboratory standard, accompanied by hypocalcemia’ (6) while we were in line with international consensus statement (7). Guidelines should thus be written to reach an audience beyond endocrinologists and/or surgeons and homogenize the definition of hypoparathyroidism.

We aimed to describe how patients with chronic hypoparathyroidism are treated. As expected, Ca salts (mainly calcium carbonate in France) and active vitamin D were the most frequently prescribed therapeutics. We showed a lower percentage of patients prescribed Ca salts (*P*  < 0.001) than previously reported (10, 12). Fifty to sixty percent of patients were treated with both Ca salts and active vitamin D, which is lower than expected (10, 12). This is probably related to a more frequent prescription of teriparatide (PTH(1–34)) in our study that can be used as an off-label drug in France. Moreover, a significant number of patients were prescribed neither Ca salt nor active vitamin D: 6–10% of patients were treated either with diuretics or teriparatide (PTH(1–34)) alone or received no specific treatment at all. Some over-the-counter treatments, such as magnesium salts, as well as dietary habits, such as consumption of a calcium-enriched diet/water, cannot be reliably evaluated by physicians alone and require patients to report what they take; this can probably explain why we observed such a difference between physician- and patient-reported rates of magnesium supplementation and why Ca salts were less used. All of these real-life data should be taken into account in studies that identify patients by their prescribed medications (12). Finally, the low rate of prescription of a combination of Ca salt and active vitamin D could be related either to milder cases or to lower adherence to guidelines.

Depending on the criteria, the adherence to international guidelines (4, 5, 7) could be graded differently: ‘high’ if we were to consider monitoring of PCa alone, which was reported to be monitored at least twice a year in 65–85% of cases or ‘very low’ if we were to consider the combination of symptoms/PCa/PPi/UCa as a composite target, which was reported in only 26–28% of cases. We acknowledge the risk of biased recruitment of physicians. Those who chose to participate in the Épi-Hypo study are probably not representative of the whole population of physicians. Their participation probably reflects their interest in the care of such patients. For this reason, their knowledge of and compliance with guidelines, although insufficient, are probably higher than that of any average French physician. Similarly, the patients who participated may not be representative of all French patients with hypoparathyroidism; they probably have a more symptomatic or difficult-to-control disease or are more concerned about the possible evolution of their disease.

The implementation of recommendations into practice depends on how they fit in with physicians’ values (14), and the most importantly identified barriers are the lack of applicability of the evidence, organizational constraints, and a lack of knowledge (15). There is currently no good approach to correctly disseminate recommendations (16), but interactive workshops might help (17). Moreover, to ensure transparency on how strongly evidence-based recommendations are, they should be rated according to the GRADE scale (18), which is how the European guidelines (4) and the latest consensus statement (7) were rated. Therefore, it is important to note that, given the very low number of clinical trials of a high level of evidence in this field, most recommendations are rated low. It is thus conceivable that those are less likely to be translated into practice. Even if guidelines formalize evidence-based knowledge on a specific topic, adhering to all the recommendations does not seem to be more effective than only adhering to some (19).

Discrepancies could exist between perceived and actual adherence to guidelines, as reported earlier by family medicine practitioners (20). Most previous surveys were based on the physician’s perspective and reported answers *via* recollection of their own habits (15, 21, 22, 23); this could be affected by recall bias. Here, we took advantage of collecting data from two sources (physicians and patients): being asked about what one usually does implies an answer either on average or by archetype. When answering questionnaires about their habits, people can tell what they most frequently do (average answer) or what they think they are expected to answer, that is socially acceptable (archetype). This is particularly highlighted by our findings on PCa values. While physicians and ePatients agreed on the definition of the PCa target values, they differed sharply from what was observed for Épi-Hypo 2019 patients. This could be explained by recall bias or a true discrepancy between the objectives and the results, highlighting how difficult it is to adequately control the disease.

Previous studies in other fields used data from medical records to study guideline compliance (14, 24). The strength of our study is to compare the global willingness of physicians for global achievements in a population of patients with chronic hypoparathyroidism. Mitchell *et al.* reported patients to be out of the targeted range for PCa in 29% of cases (10). In our cohort, we found a much larger percentage (~67%), which is explained by the definition of the target. They used a wider range (7.5–9.5 mg/dL vs 8.0–8.8 mg/dL in our study) and applying their target values, we found only 21% of our cohort to be out of this range. We wondered whether some characteristics of patients and/or physicians could explain the observed disparities in practices. We did not find any significant or consistent differences (data not shown). Importantly, most physicians agreed that both initial and continuous medical education need to be improved concerning the management of hypoparathyroidism.

Translating evidence-based medicine into evidence-based practice is important (25). To do so, it is first necessary to take into account all of the previously mentioned barriers (15) but also to use very specific wording (8, 21). Physicians are also more prone to implement-specific recommendations on issues they face in their day-to-day practice, which is important regarding data in rare diseases. Real-world data could be incorporated into guidelines to improve adherence (26), which is actually what the Épi-Hypo study aims to provide. Moreover, international guidelines are written in English while most physicians may not be fluent in English. Translating them into vernacular languages (27) will help to improve their implementation into practice; this has already been done for French-speaking countries (28). Finally, modifying chronic care also requires taking into account the understanding of patients (29, 30). We reported a gap between physicians’ and ePatients’ data regarding symptoms as a specific target; this could be either a reflection of a miscommunication in the patients-to-physician relationship or reflect a need for therapeutics to specifically target symptoms.

We therefore suggest, first, better communication from professional societies in vernacular and easy-to-understand language, which might increase the compliance to guidelines. Secondly, identifying the very physician involved in the decision-making process of a specific patient might help too; he/she could plan the care for each next coming year as well as give patient advices on which condition might require new medical evaluation. Finally, better communication between patients and physicians could minimize the perceptions gap; a longer time during visits has to be organized and could include dedicated patient-reported outcome collection.

Taken together, our data show (i) endocrinologists are part of a broad network of physicians taking care of patients with chronic hypoparathyroidism, (ii) medications vary a lot among them, especially off-label (teriparatide) and over-the-counter (magnesium salts) ones, and (iii) compliance with international guidelines regarding follow-up seems low and should be increased.

## Supplementary Material

Supplementary Materials 1

Supplementary Materials 2

## Declaration of interest

N G received consulting fees from Shire, a Takeda company, which has never been involved in any part of the present study. P K received consulting fees from Shire, a Takeda company, and Kyowa Kirin, which has never been involved in any part of the present study; he is an investigator in studies sponsored by Ultragenyx, Kyowa Kirin and Shire, a Takeda company, not related to the present study. G M has served as a research investigator for NPS Pharmaceuticals. E M received consulting fees from Medtronic that has never been involved in any part of the present study. Co S received consulting fees from Shire, a Takeda company, which has never been involved in any part of the present study. M-C V received travel grants and free meeting registrations from Pfizer, Amryt, Lilly, Ipsen, Novartis, and HRA. P H has served as an advisory board member, consultant, research investigator, and speaker for Shire, a Takeda company. J-P B received consulting fees from Shire, a Takeda company, which has never been involved in any part of the present study. The other authors have nothing to disclose.

## Funding

This work was supported by Oscar – Filière Santé Maladies Rares and the Assistance Publique – Hôpitaux de Paris (CRC 2018).
