# Supplementary material for: Practice patterns for chronic hypoparathyroidism: data from patients and physicians in France
Source: Endocr Connect. 2021 Dec 23;11(1):e210350. doi: 10.1530/EC-21-0350 (PMC8859964; doi:10.1530/EC-21-0350)
Supplement: Supplementary Materials 1 [file supplementary_material_1.pdf]

Questionnaire for ePatients.

A. In this section, we are interested in your usual follow-up in general, once your chronic hypoparathyroidism is at steady state, without any complications/symptoms:

1. How often your physician(s) check for your plasma calcium concentration? ☐<1/year ☐ 1/year ☐2/year ☐3/year ☐4/year ☐>4/year☐I have never been at steady state
2. Did your physician(s) check for kidney imagery? ☐Yes ☐No
a. If yes, how often? ☐<1/year ☐1/year ☐>1/year ☐NA
b. If yes, what kind of imagery was the most often performed? ☐Plain X-ray ☐Ultrasonography ☐ Computerized tomography ☐Other: ………… ☐NA
3. Did your physician(s) check for eye complications? ☐Yes ☐No
a. If yes, how often? ☐<1/year ☐1/year ☐>1/year ☐NA
4. Did your physician(s) check for bone mineral density? ☐Yes ☐No
a. If yes, how often? ☐only once ☐<1/year ☐1/year ☐>1/year☐NA
5. Did your physician(s) check for brain imagery? ☐Yes ☐No
a. If yes, how often? ☐only once ☐<1/year ☐1/year ☐>1/year☐NA
6. Did your physician(s) check for another morphological examen (excluding any biology)? ☐Yes : ………………………………… ☐No
7. If your chronic hypoparathyroidism is not related to surgery, did your physician propose any genetic testing? ☐Yes ☐No ☐The cause of my chronic hypoparathyroidism is surgery
8. If your chronic hypoparathyroidism is not related to surgery, did your physician ask for any family history? ☐Yes ☐No ☐The cause of my chronic hypoparathyroidism is surgery

B. In this section, we will focus on the target(s) of your treatment:

9. Were you involved into defining the aims and targets of your treatment with your physician? ☐ Yes, totally ☐Yes, partly ☐Not at all
10. Were symptoms (their absence) a target? ☐Yes ☐No ☐I don't know
11. Was the plasma calcium concentration a target? ☐Yes ☐No ☐I don't know
a. If yes, what was the target value? ☐<2.0 mmol/L (80 mg/L) ☐2.0-2.2 mmol/L (80-88 mg/L) ☐ 2.2-2.4 mmol/L (88-96 mg/L) ☐>2.4 mmol/L (96 mg/L) ☐I don't know
12. Was the plasma phosphate concentration a target? ☐Yes ☐No ☐I don't know
13. Was the calcium-phosphate product in blood a target? ☐Yes ☐No ☐I don't know
14. Was the urine calcium content a target? ☐Yes ☐No ☐I don't know
a. If yes, how often did you collect your urine for checking calcium content? ☐<1/year ☐ 1/year ☐>1/year ☐I don't remember having collected my urine for this purpose

C. The following questions are about you, personally:

15. How old are you? _ _
16. Are you ☐A man? ☐A woman?
17. Which yeat the diagnosis of your chronic hypoparathyroidism was confirmd (i.e. when a physician told you so)? _ _ _ _
18. Which year do you think your chronic hypoparathyroidism already started (i.e. the year of the surgery that causes it, if so, or the year you felt the first symptoms)? _ _ _ _
19. How many physicians (including the actual one) did you consult for finding one to take care of your hypoparathyroidism? ☐1 ☐2 ☐>3 ☐4 ☐>4
20. How far are you from the physician who follows your chronic hypoparathyroidism (answer only one way)? _ _ _ km
21. What is the actual main cause of your chronic hypoparathyroidism? ☐Neck surgery ☐ Gene defect ☐Radiotherapy ☐Auto-immune/infiltrative ☐Other: ….
22. Select the therapies you actually follow for treating your chronic hypoparathyroidism: ☐calcium-rich diet ☐Calcium supplements ☐vitamin D native (« D3 » in pills, or Uvédose®, Dédrogyl®) ☐Un-alfa® ☐Rocaltrol® ☐magnesium supplements ☐Diuretics to limit the amount of calcium in urine ☐phosphate binders (not including calcium-based oned) ☐PTH injections ☐Other: ………………………………
23. What is the specialty of the physician who follows your chronic hypoparathyroidism? …………………………………...............................
24. What is their structure to follow your chronic hypoparathyroidism? ☐For profit private ☐ Non-profit private ☐Non-university public ☐University public
25. What is the district code of the physician who follows your chronic hypoparathyroidism? _ _
26. Do you already participate in the Épi-Hypo study (www.epihypo.org)? ☐Yes ☐No ☐ I don't know
